# A roadmap to surgery in osteogenesis imperfecta: results of an international collaboration of patient organizations and interdisciplinary care teams

**DOI:** 10.1080/17453674.2021.1941628

**Published:** 2021-06-28

**Authors:** Ralph J Sakkers, Kathleen Montpetit, Argerie Tsimicalis, Thomas Wirth, Marjolein Verhoef, Reginald Hamdy, Jean A Ouellet, Rene M Castelein, Chantal Damas, Guus J Janus, Wouter H Nijhuis, Leonardo Panzeri, Simona Paveri, Dagmar Mekking, Kelly Thorstad, Richard W Kruse

**Affiliations:** a University Medical Center Utrecht, Utrecht, The Netherlands; b Shriners Hospitals for Children®-Canada, Montreal, Canada; c Ingram School of Nursing, Faculty of Nursing and Health Sciences, McGill University, Montreal, Canada; d Olga Hospital, Klinikum Stuttgart, Stuttgart, Germany; e Isala Clinics, Zwolle, The Netherlands; f Osteogenesis Imperfecta Federation Europe, Eindhoven, The Netherlands; g Care4BrittleBones Foundation, Wassenaar, The Netherlands; h Nemours/A.I. duPont Hospital for Children, Wilmington, DE, USA

## Abstract

Background and purpose — Involvement of patient organizations is steadily increasing in guidelines for treatment of various diseases and conditions for better care from the patient’s viewpoint and better comparability of outcomes. For this reason, the Osteogenesis Imperfecta Federation Europe and the Care4BrittleBones Foundation convened an interdisciplinary task force of 3 members from patient organizations and 12 healthcare professionals from recognized centers for interdisciplinary care for children and adults with osteogenesis imperfecta (OI) to develop guidelines for a basic roadmap to surgery in OI.

Methods — All information from 9 telephone conferences, expert consultations, and face-to-face meetings during the International Conference for Quality of Life for Osteogenesis Imperfecta 2019 was used by the task force to define themes and associated recommendations.

Results — Consensus on recommendations was reached within 4 themes: the interdisciplinary approach, the surgical decision-making conversation, surgical technique guidelines for OI, and the feedback loop after surgery.

Interpretation — The basic guidelines of this roadmap for the interdisciplinary approach to surgical care in children and adults with OI is expected to improve standardization of clinical practice and comparability of outcomes across treatment centers.

Expert consensus remains the best available method for guiding surgical care in most rare diseases, due to the relative lack of evidence-based practices. With a prevalence between 1:10,000 and 1:20,000, osteogenesis imperfecta (OI) is a rare genetic disease affecting the quality and quantity of collagen I. Not only bone with frequent fractures and deformities, but all tissues containing collagen I are affected (Marini et al. 2017, Chougui et al. 2020). The somewhat unpredictable phenotypic variability of the disease is often grouped according to the clinical Sillence classification I–V (Van Dijk and Sillence 2014). However, each patient is unique not only in impairments but also in treatment needs. The most severe type III has the weakest bone and not all these individuals reach the level of standing and walking. Many patients undergo surgery more than once. On the initiative of the Osteogenesis Imperfecta Federation Europe (OIFE) and the Care4BrittleBones (Care4BB) Foundation, an international interdisciplinary task force was invited to create a roadmap for a standardized, integrated approach for optimal outcomes of surgery, not only from a surgical view, but also from the patient’s perspective.

## Methods

**Figure 1.**
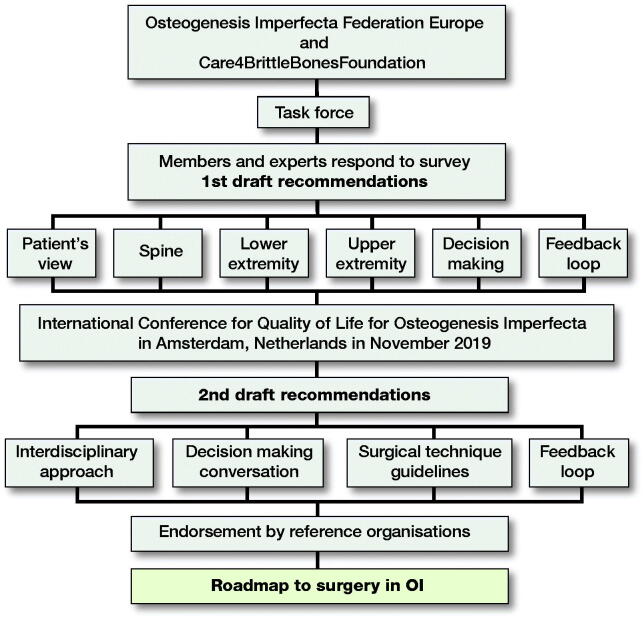
Process to develop a roadmap to surgery in osteogenesis imperfecta with international collaboration of patient organizations and interdisciplinary care teams.

The international interdisciplinary task force included members from European patient organizations and 12 healthcare professionals (HCPs) in orthopedic surgery, rehabilitation medicine, and nursing from centers recognized worldwide as leaders in the interdisciplinary care of OI. The task force developed a survey on issues around OI surgery (defined and discussed by the members) who then consulted other experts worldwide. All the responses, and the subsequent group discussions among the task force members via 9 conference calls, formed the consensus expert opinion. A set of recommendations for surgical care was then drafted and discussed at a day-long workshop during the International Conference for Quality of Life for Osteogenesis Imperfecta in Amsterdam, the Netherlands in November 2019. The recommendations were subsequently circulated to members of the Study Group on Genetics and Metabolic Diseases of the European Paediatric Orthopaedic Society and the OIFE board for endorsement (Figure 1).

## Results

4 themes were identified to guide this roadmap for surgical care in OI: the interdisciplinary approach, the surgical decision-making conversation, surgical technique guidelines for OI, and the feedback loop after surgery.

### 1. The interdisciplinary approach

**Figure 2.**
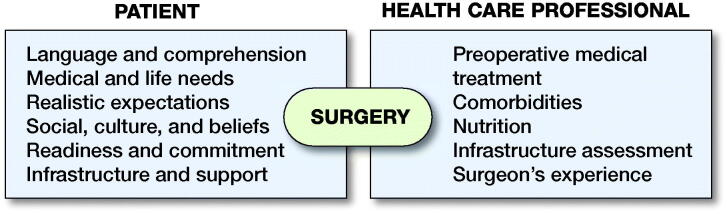
Elements of an interdisciplinary approach for surgical decision-making.

Decisions around any surgical procedure are best made involving the patient/family, the team of physicians (preferably an orthopedic surgeon, a pediatrician and/or an endocrinologist, a consultant in rehabilitative medicine, and an anesthesiologist), and other HCPs (nurses, occupational therapist, physiotherapist, psychologist, and social worker) (Figure 2).

*Patient view:*

- The patient’s ability to obtain, understand, and use healthcare information for making decisions (health literacy), and the concerns of the patient/family with regard to their functional activity limitations should be evaluated. The patient must feel free to express doubts, fears, and goals. The expected outcome from any intervention must be balanced with what is achievable surgically and how the expected and achievable results relate to autonomy, well-being, and esthetics.
- Integrate the social/cultural/belief system in the process and outcomes.
- Evaluate patient/parent compliance with regard to postoperative rehabilitation needs.
- Evaluate support, infrastructure, and home environment with regard to extended family and rehabilitation, schooling/vocational training and employment related factors. Support from an OI patient organization might be beneficial.

*HCP checklist:*

- Bisphosphonates: Optimize bone quality in growing children if needed. For adults, bisphosphonate treatment should be customized to the individual.
- Comorbidities: Any medical conditions that require monitoring should be addressed first.
- Nutrition: Strive for an optimal nutritional/metabolic status before surgery.
- Infrastructure assessment: Necessary pre- and postoperative infrastructure (medical/psychosocial/environmental) should be verified.
- Specific perioperative care for patients with OI (Rothschild et al. 2018, Beethe et al. 2020) should include:
- screening on cardiac issues (risk of mitral valve prolapse/aortic root dilatation) and chronic pain;
- careful positioning with adequate padding;
- avoid succinylcholine (fasciculations can cause fractures);
- assess for dentinogenesis imperfecta;
- use videolaryngoscopy if needed;
- avoid hyperextension of the neck;
- use adequate postoperative pain management to avoid chronic pain syndrome (higher risk in patients with OI; Beethe et al. 2020);
- consider the use of tranexamic acid (10 mg/kg bolus + 10 mg/kg/hour) for surgeries at risk of blood loss (osteotomies) and ensure X-match available.
- Surgeon’s experience: All surgeons must ask themselves if they have the knowledge and infrastructure to handle all possible complications.

### 2. The surgical decision-making conversation

Individual characteristics of the patient, the family structure, phase of life, and availability of an infrastructure for surgical care should lead to an individual approach for each case. The following key points were formulated based on the expertise and experience of the task force and experts, preliminary research, discussions in adult patient groups, and the outcomes of interviews with patients and involved family members.

- Transparent respectful partnership in shared decision-making with the patient, family (for minors), and the surgical care team.
- A thorough evaluation of fractures, deformities, and functional needs; and a structured questionnaire or interview to explore the importance of the goals, patient’s satisfaction, and expectations are needed (Law et al. 1990).
- Find support and information from specialized consults, second opinions, and patient advocacy groups in case the patients/family’s goals, expectations, and priorities cannot be matched.
- Decision-making drivers and structures might vary in cultural environments with different values, including the role of the decision-maker. If necessary, use a professional cultural interpreter/translator to include the patient’s cultural values and the local regulations in the informed consent procedures.
- The final decision taken by the patient/family should be respected. In cases of harm and neglect, the appropriate pathways should be followed.

### Surgical technique guidelines for OI: Disease specific details

#### General

**Figure 3.**
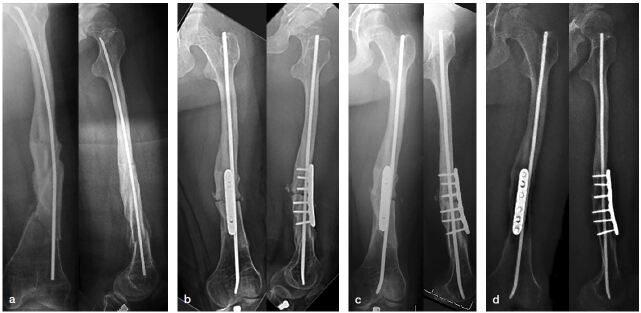
a. 15-year-old female with OI type III, treated with bisphosphonates, ambulatory with multiple fractures over a pre-existing flexible nail. b. Lateral closing wedge at the CORA, 4.5 mm flexible nail together with 6-hole 3.5 mm locking plate for length and rotational stability. c. Union without recurrent fractures or deformity at 2 months’ follow-up. d. Union without recurrent fractures or deformity at 2 years and 5 months’ follow-up.
- Avoid plates/screws as stand-alone implants to avoid stress fractures at the edges of the plates/screws.
- Consider bone grafting for any bony defects. Allograft bone is preferable over autogenous graft to preserve the maximum amount of bone.
- Immobilization postoperatively in backslab or plaster cast is followed by initiation of mobilization as soon as healing permits. Children with no previous experience ambulating may require some bracing and/or walking aids. In adult patients who have solid intramedullary nailing after transverse fractures or osteotomies, full weight-bearing is allowed without the use of a brace or plaster. Oblique fractures with non-solid fixation can be treated with partial weight bearing with or without brace or plaster.

- Use single or multilevel multidirectional osteotomies at the apices of deformities of long bones and implantation of intramedullary (IM) rods for stabilization.
- For acute fractures, IM implants are preferred for fixation after closed or open reduction.
- Avoid oversizing rods to avoid stress shielding and subsequent bone loss. Elongating implants or constructs for stable longitudinal growth are usually preferred. Fixed-length devices can be used as an alternative, especially in adults or when bone size is small or lengthening devices are not available.
- Use bony shortening with correction of deformities.
- Occasionally additional soft-tissue release or muscle lengthening is necessary to allow correction of excessive contractures.
- Closed osteoclasis might minimize blood loss and periosteal disruption.
- Consider supplemental plating of bones after IM nailing for rotational control and non-unions, especially in older patients (Figure 3).

*Lower extremity (LE) surgery in OI*

- Recurrent fractures, anticipated progressive deformity, and/or expected improved function and ambulation after surgery are indications for surgical alignment and increasing bone stability with IM implants (Figure 4). Figure 4.a. Preoperative radiographs of a 6-year-old female with OI type III with bilateral antero-lateral deformity of the femur and tibia and tibial pseudoarthrosis. Repeated fractures, inability to weight bear or ambulate with unaffected upper extremities indicated candidacy for IM rodding.b. Postoperative radiographs. Femur: correction of malalignment with telescoping rods. Proximal female threads are inserted in greater trochanter not crossing the trochanter apophysis.Tibia: female threads are positioned into the proximal tibial epiphysis, not crossing the physis. Threads of the male rod are inserted in the distal tibia epiphysis not protruding into the ankle.

**Figure:**
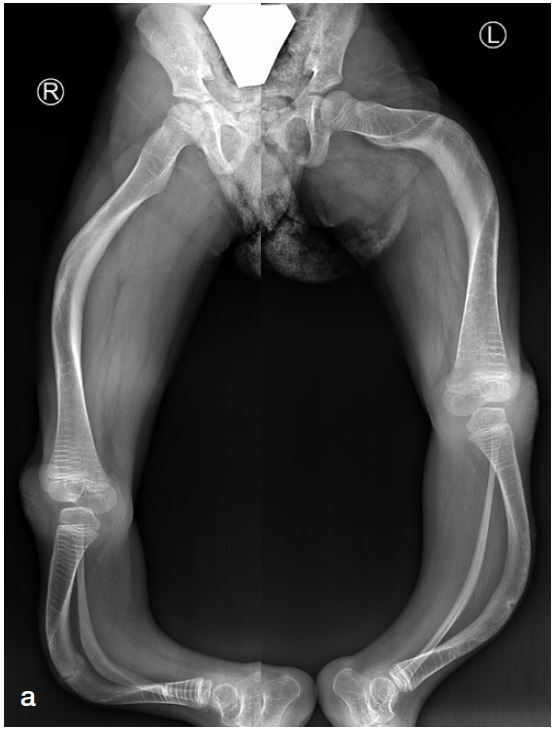

**Figure:**
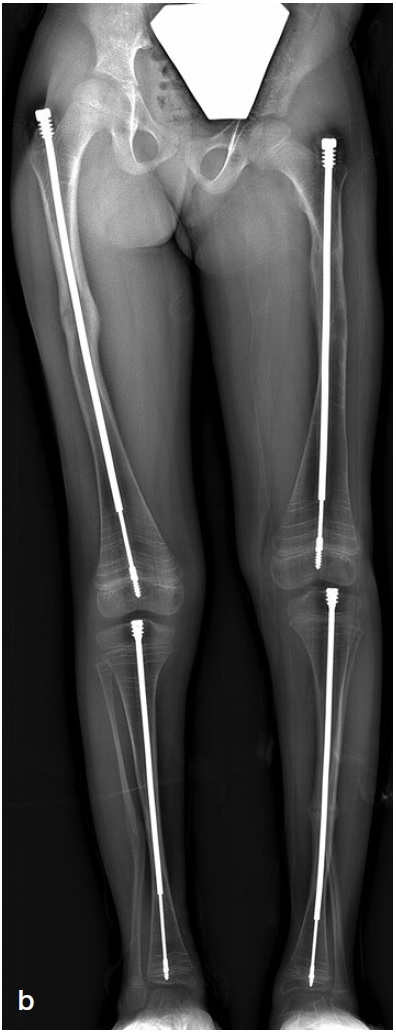
- Operating maximal 2 bone segments in the same session is preferred by most surgeons.

*Upper extremity (UE) surgery in OI.*

**Figure 5.**
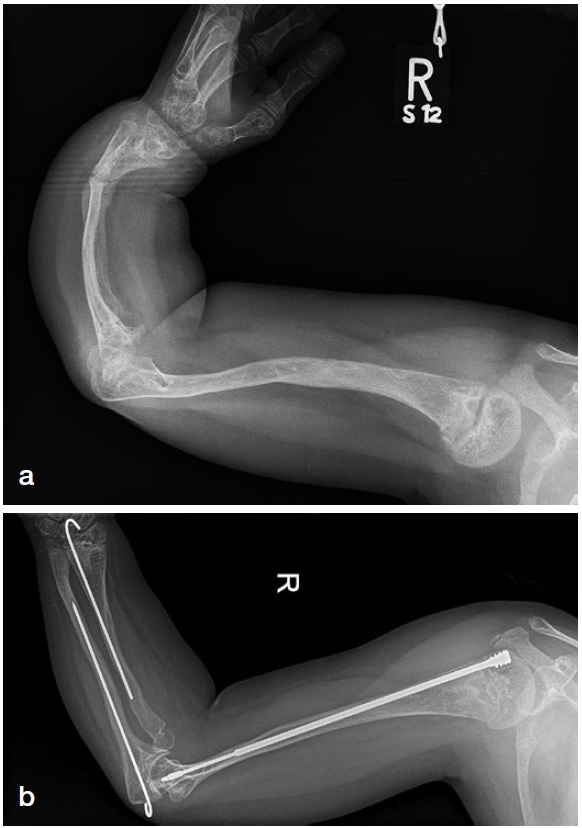
a. Severe deformity of the upper extremity in an 8-year-old patient with OI type III. Double osteotomy of the humerus and telescopic rodding was planned plus a double corrective radius and ulna osteotomy with a K-wire fixation inserted from opposite sides through the growth plates, thus allowing for telescoping. b. Postoperative follow-up at 5 years. The amount of telescoping of the nail in the humerus corresponds with length without K-wire in radius and ulna due to growth.
- With radial head dislocation, operate only for major impairments, severe pain, or if function is markedly limited.

- In case of deformity in both the humerus and forearm, correct and stabilize the humerus first before correcting the forearm.
- When bilateral surgery is indicated, a “one-side-after-the-other” approach is good practice.
- The distal humerus always needs a 3-dimensional correction. If the construct lacks stability, one option is to immobilize the extremity for 3–4 weeks in a plaster to keep the correction during consolidation.
- Sandwich constructions with allograft are advised for humeri with a very small diaphyseal diameter and non-unions.
- In forearm surgery, the application of K-wires from opposite directions (antegrade in the ulna, retrograde in the radius) allows for better stabilization during growth (Figure 5).

*Spine surgery in OI*

- Evaluate occiput C1–C2 anatomy before any surgical treatment of the spine.
- 3D planning of the spine with CT scan is helpful for surgical planning.
- A Cobb angle of 45°–50° is usually an indication for surgery. In severe OI, early surgery with Cobb angles still under 40° might be advisable as progression of scoliosis will most likely occur. Surgery is relatively easier for the patient and the surgeon, with better outcomes and lower risk of complications.
- Pulmonary function is often compromised in scoliosis and should be measured longitudinally.
- Fusion of at least the entire curve should be considered to prevent progressive residual deformity outside the fused segment.
- Decision-making for including pelvic fixation (or not) should include the preoperative ambulatory status, presence or not of L5 pars fracture (spondylolisthesis), and quality of the distal fixation.
- Preoperative (4–12 weeks) and intraoperative traction (intraoperative includes cranial and skin leg traction) can optimize deformity correction and reduce the stress on the spinal implants during surgical reduction.
- Cranial traction when used should involve 6–10 pins.
- Augmentation of the construct using sublaminar wires can help reduce the stress over pedicle screws, or the wires add fixation on points with impossible pedicle screw insertion (Figure 6). Figure 6.a. Preoperative radiograph of a 15-year-old patient with OI type III with progressive scoliosis.b. Note the decreased space for pedicle screws in the codfish vertebrae and increased diameter of the intervertebral disks. Augmentation with a sublaminar wire at level L4.

**Figure:**
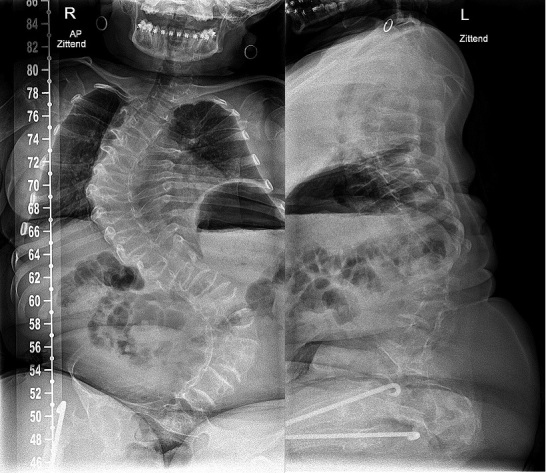

**Figure:**
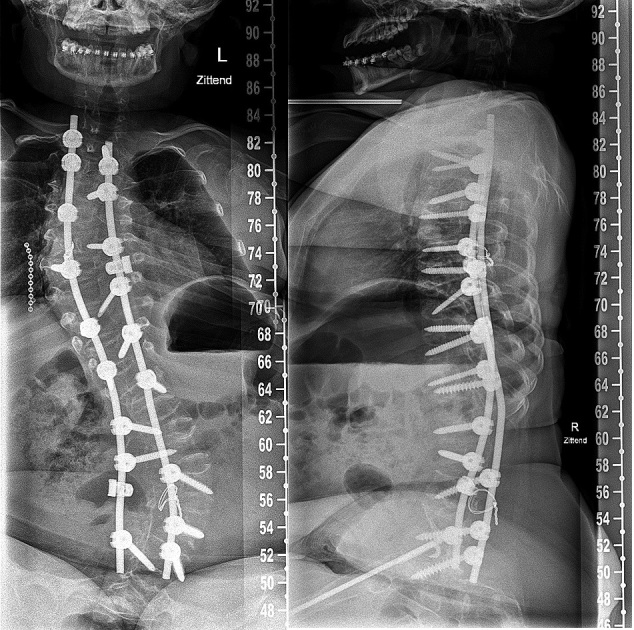
- Use of a postoperative brace (6–12 weeks) might be considered.
- In symptomatic cranio-cervical deformities, neurological structures should be decompressed, and occipital-cervical fusion should be done. Realignment and fusion for basilar invagination is usually done through a posterior approach. If symptomatic spinal cord compression persists, additional direct anterior decompression might be considered.

### Feedback loop after surgery (Figure 7)

Pre- and postoperative standardized outcome measurements to link the expected outcomes to the actual outcomes in a feedback loop not only measures the success of patient and clinical outcomes, but also regularly monitors the efficiency and effectiveness of the team’s processes and practices and contributes to global research efforts to continuously establish best practices.

**Figure 7.**
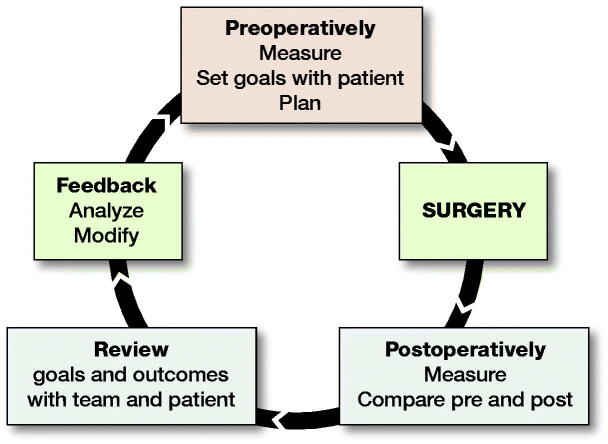
Feedback loop for OI surgery.

A feedback loop with both clinically reported and patient-reported outcomes should include:

- Preoperatively: standardized measurements of deformities, pain, function, and quality of life (QoL), and realistic goals and expectations with the patient/family.
- Postoperatively: reassessment of deformities, pain, function, QoL for comparison with preoperative measures, and a determination of whether goals were met. Time should be taken to discuss with the patient his/her overall satisfaction and compare this with the views of the surgical team.
- The recently published Key4OI Standard Set of Core Outcome Measures for OI is highly recommended to ensure global research is possible (Nijhuis et al. 2021).

## Discussion

These recommendations for the surgical care of patients with OI, recognizing both patient and HCP concerns, represent a summary of the expert opinion of an international task force of 15 “OI experts,” including representatives from patient organizations.

For optimizing bone quality, bisphosphonates have routinely been used in patients with OI in the last 20 years (Dwan et al. 2014), and a consensus for their use in children and adolescents was recently published (Simm et al. 2018). Positive and negative effects of bisphosphonates on fracture rate, osteotomy healing, curve behavior in scoliosis, and cranial base pathology, and the effect of discontinuation of bisphosphonates around surgery, remain a topic for discussion due to possible study design biases (Anissipour et al. 2014, Dwan et al. 2014, Ng et al. 2014, Arponen et al. 2015, Simm et al. 2018, Ralston and Gaston 2020). Nevertheless, the task force agreed that ensuring optimal bone quality is critical for surgical intervention and treatment should be tailored to the surgical plan. Bisphosphonate regimes should be different in a growing skeleton compared with treating an adult skeleton (Brizola and Shapiro 2015, Simm et al. 2018).

Health literacy assessment varies in different parts of the world, due to diverse social cultures and educational levels. Understanding the social and cultural backgrounds of the patient/family, avoiding violation of societal norms as much as possible, is considered paramount. The hierarchy in families, specific gender roles in different cultures, social acceptance of disease, and attribution of disease to various non-medical causes are important factors (Kahissay et al. 2017). Thresholds for patients/families to seek medical help as a result of these factors might be a major influence in the surgical process. When physicians or the primary sources of medical care are not available, effective primary medical care might be organized by mothers, elders, or alternative medical practitioners.

Surgery-specific guidelines for OI are focused on disease-specific fractures, deformities, and pain and should lead to improvement of function, participation, and QoL. The severity of bony deformity in the lower limb is associated with fracture risk (Caouette et al. 2014). Adequate timing of surgery for deformities of the skeleton is always a challenge. There are no distinct age limitations on surgery. Lower extremity rodding is often indicated when a child with significant bowing attempts to stand. Operating on children less than 2 years old with small bone size is technically demanding and may result in complications but may be indicated due to a high fracture rate. The number of long bones that are operated on in one session should depend on the strategy and implants that work in the surgeon’s infrastructure. In the growing skeleton, telescoping rods are usually preferred over solid rods. However, the frequency of reoperation due to rod migration and telescoping failure has not improved much in recent decades. To date, revision rates, between 30% and 50%, and re-revision rates around 30% within 5 years of follow-up, have been reported with the current elongating devices (Azzam et al. 2018).

Upper extremity surgery in OI should specifically focus on self-care activities (dressing, hygiene), daily activities (propelling wheelchair or walker, computer access) and participation in school/work/leisure life for increased autonomy of the individual (Khoshhal and Ellis 2001, Mueller et al. 2018). Prior to UE surgery, evaluation by an occupational therapist can assist in identifying strengths and impairments. This is useful for clarifying surgical goals. Upper extremity surgery in OI is more demanding due to the size of the bones, making them more difficult to align with intramedullary devices (Wirth 2019). The use of assistive devices or environmental modifications could resolve functional problems and should be tried prior to surgical options.

The interdisciplinary team approach as described is especially indicated in the management of spinal disorders in OI. Progressive scoliosis, cranio-cervical deformities, and spondylolisthesis are the most common spine deformities in OI for which ample experience and training is needed (Wallace et al. 2017, Castelein et al. 2019). One of the indications for scoliosis surgery is to prevent deterioration of lung function. Yet the correlation between scoliosis (Cobb angle) and pulmonary function is known to be weak and OI intrinsic factors and chest wall deformations play a more important role (Bronheim et al. 2019). The risk for progression of scoliosis in hyperlax patients remains unclear (Engelbert et al. 1998).

Basilar invagination is the most common cranio-cervical deformity in patients with OI (Arponen et al. 2015). Sleep studies can help detect nocturnal episodes of apnea in cases with potential brain stem compression. Surgery increasingly plays an important role, but controversy remains as to whether asymptomatic patients with radiological basilar invagination should be operated on to prevent neurology (Wallace et al. 2017, Castelein et al. 2019).

In general, adaptability of the surgical plan, skill, and experience in the wide variability of OI is critical. Defining “success” is patient specific. Perceived improvement by the patient, and measurable improvement, should include objective clinical measures, patient-reported measures, and goal-attainment tools (Kiresuk et al. 1994) to implement a feedback loop for evidence-based care improvement. The new Key4OI Standard Set of Core Outcome Measures for OI was designed for this purpose and its broad implementation will allow comparative research and value-based healthcare reform (Nijhuis et al. 2021). Quantitative outcome measures and qualitative insights should be weighed in defining improvement or success.

## Conclusion

This roadmap to surgery in OI, initiated by and created with patient organizations and in collaboration with international interdisciplinary expert care teams, is a set of guidelines for optimizing surgical care in OI.
